# Perceptions and reported practices of pregnant women and mothers of children under two years of age regarding antibiotic use and resistance in Vientiane province, Lao PDR: a qualitative study

**DOI:** 10.1186/s12884-022-04894-7

**Published:** 2022-07-16

**Authors:** Vanphanom Sychareun, Paphatsone Phounsavath, Amphoy Sihavong, Sengchanh Kounnavong, Kongmany Chaleunvong, Anna Machowska, Bounxou Keohavong, Mayfong Mayxay, Jaran Eriksen, Claudia Hanson, Manivanh Vongsouvath, Annelie Brauner, Jo Durham, Cecilia Stålsby Lundborg

**Affiliations:** 1 Faculty of Public Health, University of Health Sciences (UHS), Vientiane, Lao PDR; 2 Lao Tropical & Public Health Institute, Ministry of Health (MOH), Vientiane, Lao PDR; 3 Vientiane Capital Health Department, MOH, Vientiane, Lao PDR; 4 Institute of Research and Education Development, UHS, MOH, Vientiane, Lao PDR; 5 Department of Global Public Health, Karolinska Institutet, Stockholm, Sweden; 6 Food and Drug Department, MOH, Vientiane, Lao PDR; 7 Lao-Oxford-Mahosot Hospital-Welcome Trust Research Unit (LOMWRU), Microbiology. Laboratory, Mahosot Hospital, MOH, Vientiane, Lao PDR; 8 Department of Microbiology, Tumor and Cell Biology, Karolinska Institutet, Stockholm, Sweden; 9 Faculty of Health, Centre for Healthcare Transformation, School of Public Health and Social Work, Queensland University of Technology, Brisbane, Australia; 10 Centre for Tropical Medicine and Global Health, Nuffield Department of Medicine, University of Oxford, Oxford, UK; 11 Department of infectious diseases, Stockholm South General Hospital, Stockholm, Sweden

**Keywords:** Antibiotic use, Antibiotic resistance, Pregnant women, Mothers, Perceptions, Practices, Lao PDR

## Abstract

**Background:**

Understanding pregnant women and mothers’ perceptions towards antibiotic use and resistance is essential for appropriate antibiotic use and limiting antibiotic resistance. This study aimed to explore perceptions and reported practices of pregnant women and mothers with children under two years of age regarding correct antibiotic use and antibiotic resistance in Vientiane Province, Lao PDR.

**Methods:**

The study employed an exploratory qualitative research design using focus groups discussions (FGDs). Participants were purposively selected based on: being pregnant at third trimester and attending antenatal care and mothers with children under two years of age, attending the health facility for postpartum visit /vaccinations. Six focus group discussions were conducted in September 2019 with a total of 55 women. The FGDs were transcribed verbatim, data were analyzed first by coding then categorizing the data as we looked for patterns and themes by using the qualitative content analysis.

**Results:**

Most participants had some understanding of antibiotics but wrongly believed antibiotics can be used to treat viral disease. Over half of the participants had heard the term “antibiotic resistance”, but often believed it was their bodies, not the bacteria that developed antibiotic resistance. During pregnancy and for their infants, women preferred to use antibiotics only when prescribed by a doctor. Outside of pregnancy however, consuming antibiotics without a prescription was commonly reported. Participants wanted more information about the indications for antibiotic use and antibiotic resistance.

**Conclusions:**

More effort is required to increase the level of understanding, and practice of mothers to promote optimal antibiotic use. Mothers’ desire to learn more, and their fundamental concern for their children, can be used to promote appropriate antibiotic use. Awareness raising should be complemented by efforts to address other determinants of inappropriate antibiotic use, including educating healthcare workers, and pharmacists and addressing health service determinants that contribute to inappropriate antibiotic use.

## Background

Pregnancy is a period of great physiological changes with pregnant women typically more susceptible to bacterial infections than non-pregnant women [1, 2]. Common bacterial infections during pregnancy can be treated with antibiotics which can prevent potential complications associated with infections in pregnancy, such as miscarriage, stillbirth, preterm rupture of membranes and preterm birth. Taking antibiotics during pregnancy can also have short- and long-term negative effects (e.g., change in gut microbiome, which potentially increases the risk of developing asthma and atopic dermatitis among children) in newborns [3–11]. Antibiotics are also commonly prescribed medications for children younger than two years of age, as the most important modality for treating and preventing infections [12]. The overuse of antibiotics in children, however, can place them at risk of future chronic diseases and allergies [6, 13].

Inappropriate use of antibiotics during pregnancy and in children under two years of age, is also considered an important contributor to antimicrobial resistance, an issue of global concern [14, 15]. Inappropriate use of antibiotics is influenced by multiple factors, including availability of diagnostic testing and appropriate antibiotics in health system, infection prevention and control and prescribing practices [14]. At the individual level, therapeutic preferences, knowledge and attitudes towards antibiotics, are important drivers of antibiotic use [2]. Individual factors influencing inappropriate use include erroneous beliefs such as antibiotics are effective against viruses, or once symptoms have resolved, treatment can be discontinued without completing the prescribed antibiotic course [16–20]. Studies have demonstrated that pregnant women may discontinue antibiotic treatment after the confirmation of pregnancy due to perceived negative health and teratogenic risks of antibiotic use [21, 22].

In low and lower middle-income countries, inappropriate antibiotic use is also often facilitated by over the counter availability of antibiotics [17–20, 23–27]. Use of antibiotics is critical for severe pediatric infections [12]. Self-medication with antibiotics as commonly observed in many low and middle-income countries, and unnecessary medications including inappropriate antibiotic use can however cause increased risk of antibiotic resistance, treatment failure, and increased healthcare costs [6, 28]. Thus, the use of antibiotics by the most vulnerable populations - children and pregnant women - has to be carefully monitored and evaluated bearing in mind side effects such as nausea, diarrhea or quinolone toxicity to the developing skeletal system [29]. Understanding the population’s awareness and understanding of antibiotic use and resistance is recognised as critical in developing effective interventions to reduce inappropriate use [24, 30, 31].

The Lao People’s Democratic Republic (Lao PDR) is a lower middle-income country situated in South East Asia. Information about understanding of antibiotic use and resistance is scant, but there is evidence of inappropriate antibiotic use. Studies suggest that purchasing antibiotics over the counter without a prescription is common [25, 32–34]. A recent study conducted among healthcare providers (HCPs) regarding antibiotic use and resistance in relation to pregnancy, childbirth, and children under two in Lao PDR showed that two-thirds of participants reported non-indicated antibiotics prescribing for uncomplicated vaginal delivery, and half of HCPs did not believe that their prescribing practice contributed to antibiotic resistance [33]. Antibiotic prescription for under fives with a common cold or urinary tract infections has also been reported as common, as has poor adherence to the antibiotic regimen [35] .

Inappropriate antibiotic use during pregnancy and infancy may put the mother and child at greater risk for maternal and child adverse outcomes as well as increased antibiotic resistance. Studies on perceptions and reported practices of pregnant women and mothers of children under two regarding antibiotic use and resistance is lacking in Lao PDR. Therefore, it is critical to understand pregnant women and mothers’ perceptions towards antibiotic use and resistance in order to improve appropriate antibiotic use, especially among these vulnerable groups.

The purpose of this study was to explore perceptions and reported practices of pregnant women and mothers of children under two regarding antibiotic use and resistance in Lao PDR. This study could provide useful information for future intervention aiming to improve appropriate antibiotic use contributing to contain antimicrobial resistance.

## Methods

### Study design

An exploratory qualitative research design using focus groups discussions (FGDs) was chosen to obtain the rich, in-depth information needed to understand and interpret knowledge, experiences and practices of pregnant women and mothers having children two years of age. Group discussions can generate in-depth understanding of participants’ experiences and beliefs and allow for an exploration of multiple dimensions of an issue under examination [36, 37] This study is a part of the CAREChild (Containment of Antibiotic REsistance—measures to improve antibiotic use in pregnancy, childbirth, and young children) project conducted in Lao PDR [29].

### Study setting

The study setting was Thoulakhom district in Vientiane Province. Vientiane Province has a population of 419,000 inhabitants and has 11 districts. Thoulakhom district, with a population of 57,791 inhabitants [38], was purposively selected for this study due to its diverse socio-demographic background. The district has one district hospital and five health centers and covering 21 villages [38]. Most of the population have relatively easy access to a pharmacy and 71% live within 10 km of a district health facility or community health centre. The FGDs were conducted at one district hospital and two health centers.

### Selection of participants

Participants were purposively selected based on being (i) pregnant at third trimester attending antenatal care (ANC), and (ii) mother with children under two years of age, attending the health facility. Pregnant women were identified via the local health ANC registry and invited to participate during their ANC visit if they gave informed consent. Mothers who participated in FGDs were identified during a postpartum visit and/or child vaccination visit and approached by local health staff who informed potential participants of the study and its objectives. All health facilities including district hospital and health centers in Thoulakhom district were selected using simple random sampling based on the health facility name list available at the district health office.

### Data collection

At each selected health care facility, two FGDs were conducted, one with pregnant women and one with mothers with children under two years of age. Three groups of pregnant women in the third trimester with a total of 29 participants and three groups of mothers with children under two years of age with a total of 26 participants were conducted. Each FGD consisted of eight to eleven participants with varying occupations, age, education, number of pregnancy and children background. Altogether, six FGDs with a total of 55 participants were included.

 A semi-structured interview guide for FGDs was developed and pretested among one group of pregnant women at third trimester with eight participants and one group of mothers of children under two with eight participants at Phonehong district (Vientiane province), with similar characteristics as the target population. Participants were asked about their perceptions towards antibiotic side-effects and resistance, consequences of antibiotic resistance, experience with antibiotic use, information seeking and preferred sources of information. Based on the feedback from the pretest, the interview guide was adjusted and finalized.

The FGDs were guided by three of the researchers (one facilitated the FGD, another took notes and the third was the supervisor). The FGDs were conducted at the meeting room of the district hospital or health centers. Each FGD lasted 60–90 min and was closed when no new ideas were emerging. Participants were coded according to parity status (pregnant women and mothers with children under two years of age). Each participant received a small gift of towel to thank them for their time.

### Data management and analysis

The audio records and notes of the discussions were transcribed verbatim. The Lao transcripts were translated into English and manually analyzed using manifest and latent qualitative content analysis [37, 39]. We applied the manifest analysis which deals with the visible and obvious components, and the latent analysis which is denoted as interpretation of underlying meaning of the text and deals with the relationship aspect. Three authors (VS, AS and PP) did the main analysis with additional contributions by other co-authors. The transcripts of the discussions were read through several times to obtain a sense of the whole. Meaning units were identified, condensed and coded. During the coding procedure, the Lao and English versions were used simultaneously to avoid misinterpretations of the full meaning of the texts. The various codes were compared based on differences and similarities of perceptions, then sorted into sub-categories and categories. Finally, the authors reflected and compared the findings between the different FGDs to reach agreement on the codes, categories and subsequent themes. Two themes emerged; ‘Perceptions of antibiotics and antibiotic resistance’ and ‘Reported practices of antibiotic use and information seeking’ (Table 1).

**Table 1:** Categories and themes of the analysis

Categories	Themes
Perceptions of the potential side-effects of antibiotics	Perceptions of antibiotics and antibiotic resistance
Perceptions of antibiotic resistance	
Perceptions of the consequences of antibiotic resistance	
Reported practices of antibiotic use	Reported practices of antibiotic use and information seeking
Information seeking	
Preferences for antibiotic and antibiotic resistance information	

The validity of the analysis was ensured by: (1) the triangulation of information; (2) coding undertaken by three researchers (VS, AS and PP) independently, with differences discussed between coders to reach agreement. In reporting the findings, codes are used to maintain anonymity of the key informants.

### Ethical consideration

Ethics approval was obtained from the National Ethical Committee for Health Research, Lao PDR (Ref. NECHR No. 031 /NECHR, dated 27th March/ 2019). All methods were carried out in accordance with relevant guidelines and regulations. In addition, prior to undertaking the study, the study design and purpose were discussed with the head of each community health center and their approval gained. Participants and the researchers did not know each other, but participants were aware of the interviewers’ status as researchers. Verbal informed consent was gained from each participant after an explanation of the study objectives, assurance of confidentiality and that choosing not to participate would not disadvantage them in any way.

## Results

### Background characteristics of the participants

Overall, 29 pregnant women and 26 mothers of children under 2 years of age participated in six FGDs. The mean age of the pregnant women was 27 years (range 17–40), most were housewives and rice farmers, slightly more than half (17/29) had completed primary education (Table 2). The mean age of mothers with children under 2 years was 26 years (ranged from 17 to 35 years) and most were rice farmers (11/26).

**Table 2:** Socio-demographic characteristics of FGD participants

No. FGD	No. of partici-pants	Category of participants	Age range	Range of No of pregnancy and delivery	Occupation	Education
**1**	9	Pregnant women at 3rd trimester	22–29	1–3 and 0–2	8 housewives 1 student	2 not completed primary school. 4 primary school 3 secondary school
**2**	11	Pregnant women at 3rd trimester	19–40	1–6 and 0–5	7 rice farmers 3 housewives 1 trader	3 not completed primary school. 7 primary school 1 secondary school
**3**	9	Pregnant women at 3rd trimester	17–39	1–4 and 0–2	4 rice farmers 5 housewives	2 not completed primary school. 6 primary school 1 secondary school
**4**	8	Mothers with children < 2 yrs old	19–35	1–2 and 1–2	5 rice farmers 2 housewives 1 worker	2 not completed primary school. 5 primary school 1 secondary school
**5**	9	Mothers with children < 2 yrs old	17–33	1–3 and 1–3	3 rice farmers 6 housewives	3 not completed primary school. 5 primary school 1 secondary school
**6**	9	Mothers with children < 2 yrs old	22–31	1–3 and 1–3	3 rice farmers 6 housewives	2 not completed primary school. 7 primary school

### Perceptions of antibiotics and antibiotic resistance

#### Perceptions of the potential side-effects of antibiotics

Most participants were aware that antibiotics can have side-effects with anaphylaxis as the most commonly cited. Some recounted experiences of breathing difficulties after taking Ya-am-pi (ampicillin) or skin rashes:

*“I took antibiotics, and I had some adverse event such as feeling sleepy. I was allergic to ampicillin injection which caused respiratory distress and dyspnea”* (FGD#4 with mother, respondent#6, 31 years old).

One pregnant woman thought taking antibiotics during pregnancy may lead to congenital malformation. Other negative side-effects reported by participants included feeling weak, fatigue, vertigo and weight loss in pregnant women. A few participants thought that taking too many antibiotics could dry the womb out, resulting in miscarriage and infertility:

“*If the pregnant women have overconsumption of antibiotics, their pregnancy could have been aborted. The older people were told not to take too many antibiotics, but we don’t know how bad it is. I thought that it could affect t the bone.”* (FGD#2 with pregnant woman, respondent #9, 22 years old).

*“The side effect of antibiotic was that if women take too much antibiotics in a situation where it is not needed, the uterus can be dry, they could be thin, making them less likely to get pregnant or infertile”* (FGD#1 with pregnant woman, respondent #1, 29 years old).

Another reported side-effect was fatigue. Some mothers felt taking antibiotics after delivery could decrease breast milk production and cause a dry throat.

*“I thought that the adverse event was like the side effects of antibiotics overconsumption where it would make you thin, dry throat and no breast milk”* (FGD#6 with mother, respondent#5, 31 years old).

#### Perceptions of antibiotic resistance

 Over half of the participants had heard the term “antibiotic resistance” or in Lao language as “ Kan tan to ya tan xeua” which they described as a condition whereby infections could not be cured by previously used antibiotics, requiring a change of antibiotics. Typically, women understood it was their bodies, not bacteria, that developed antibiotic resistance. Understanding of antibiotic resistance was also informed by the women’s’ experiences as illustrated below:

“*Antibiotic resistance is taking antibiotics without a prescription from the doctor in which the medicine works effectively at the beginning but fails the second time. My son often had pneumonia and went for check-up. The doctor prescribed antibiotics, but it was not effective. Later, the doctor tried to prescribe a different antibiotic and my son recovered.”* (FGD#6 with mother, respondent#6, 26 years old).

Some mothers cited a link between antibiotic resistance and superbugs. Examples women gave of antibiotic misuse included substituting the prescribed antibiotic for a different one, inappropriate use of antibiotics, taking antibiotics for only mild symptoms or self-medicating. The following quotes highlight some of the mother’s understanding of how antibiotic resistance is caused:

*“Whenever people got sick and take an antibiotic by themselves without doctor’s prescription, they will become antibiotic resistance*” (FGD#5 with mother, respondent#5, 22 years old) *“Antibiotic resistance is the overconsumption of the antibiotic, but it is not effective anymore and they need to change to a new type of antibiotics. If the patients follow the doctor’s advice and completed the antibiotic treatment, there will not be a medicine resistance”* (FGD#6 with mother, respondent#4, 30 years old).

Not all participants understood the meaning of antibiotic resistance however, illustrated in the quote below:

*“I had no ideas regarding the antibiotic resistance. Our family did not usually use the antibiotics unless we have some wounds”* (FGD#2 with pregnant women, respondent#3, 25 years old).

#### Perceptions of the consequences of antibiotic resistance

Most participants recognized antibiotic resistance could harm human health, as one participant explained:

*“Resistance to the antibiotic had impact to the human body (e.g. body weakness, fatigue…) leading to severe complications and some fatalities. However, the consequences of antibiotic resistance were the physical deterioration which can be life threatening”* (FGD#4 with mother, respondent#4, 29 years old)

Most participants understood antibiotic resistance makes it harder to treat some infectious diseases and felt it could lead to adverse effects, such as prolonged fever, weakness, loss of appetite, and increasing severity of the condition being treated. One pregnant woman explained that antibiotic resistance could lead to recurrent infections:

*“The consequences of antibiotic resistance were whenever we had pharyngitis, pneumonia, the patients took antibiotic orally, which could not be cured, so they will change to antibiotic injection. The consequences of antibiotic resistance will keep the infections reoccurring”* (FGD#1 with pregnant women, respondent#2, 27 years old)

One mother linked antibiotic resistance to potential side-effects such as rashes and respiratory distress:

*“Some patients took amoxicillin or ampicillin wrongly which they could have an allergic, and trouble breathing, and I think that this is the consequence of antibiotic resistance”* (FGD#4 with mother, respondent#1, 29 years old)

### Reported practices of antibiotic use and information seeking

#### Reported practices of antibiotic use

Most participants said that they preferred not to take antibiotics while being pregnant. When not being pregnant however, several reported buying antibiotics from private pharmacies to treat the common cold, pharyngitis, or fever. Commonly used antibiotics were amoxicillin or ampicillin. Some participants said they had taken antibiotics for a headache, based on blood test results which showed increased level of white blood cells, dysuria, and leucorrhea, skin lesion, after abortion, after normal vaginal delivery or after caesarean section. Some women had also been prescribed antibiotics by their doctor while being pregnant:

*“I had vaginal bleeding while being pregnant in the 4th month. I was admitted in the hospital, and I got the intravenous (IV) line. The doctor also gave me 1 flacon of antibiotics through IV. Usually, I took amoxicillin or ampicillin and paracetamol.”* (FGD#4 with mother, respondent#3, 22 years old)

*“The doctor gave antibiotic to patients when suturing the caesarean section, vaginal bleeding while being pregnant or abortion after discharging from the hospital.”* (FGD#4 with mother, respondent#7, 30 years old)

Participants expressed that they were somewhat reluctant to take antibiotics during pregnancy, concerned about the risk of congenital malformation. This might also explain non-adherence to antibiotic regimens, as below:

*“I had been taking antibiotics when I was 3–4 months pregnant due to heavy headaches and vertigo. I went to the army health clinic then I was diagnosed that the leukocytes in blood vessels had risen and the doctor prescribed ampicillin for 7 days. Though I stopped on the 3rd day because the symptoms were recovered well.”* (FGD#2 with pregnant women, respondent#5, 33 years old)

The women felt however that children should be given antibiotics to fasten recovery.

“*There is an advantage of using antibiotic to kids, it is treating their sickness such as pneumonia. Last time I brought my kid to Mittaphab hospital, doctor prescribed antibiotic and my kid recovered within three days*.” (FGD#6 with mother, respondent#2, 17 years old)

Common reasons for giving antibiotics to their children, were fever, cough, pneumonia or pharyngitis. Amoxicillin, ampicillin, and erythromycin were the most used antibiotics. Some mothers also said they kept some in the house in case of illness.

*“My kid took antibiotics when he had pharyngitis or pneumonia. I bought antibiotic in the form of powder that costed about 10,000 LAK, but this medicine was not effective, however, the antibiotics from overseas that costed 40,000 LAK were more effective*” (FGD#6 with mother, respondent#8, 22 years old)

Note: 1US dollar = 10,000 LAK.

#### Information seeking

Most participants received information about antibiotics and antibiotic resistance from healthcare providers in public hospitals, private clinics and pharmacies and trusted the advice of the allopathic doctors:

*“I understand what the doctors said, and I believe in the decisions of doctors because they are well educated in this field. I have heard some doctors said that the infectious diseases will last longer if we don’t follow doctor’s advice to take antibiotic correctly. Pneumonia will take longer time to be treated.”* (FGD#5 with mother, respondent#6, 28 years old)

Other sources of information related to antibiotic use were neighbours, friends, and family with experience in taking antibiotics. A few mothers said they had heard about antibiotics and antibiotic resistance from television and the internet.

*“I knew about antibiotic from my relatives as she used the antibiotic for her children*” (FGD#6 with mother, respondent#7, 24 years old)

*“I heard about using antibiotics from my friend during her pregnancy as the doctor prescribed antibiotic to her when she got some infectious diseases”* (FGD#3 with pregnant women, respondent#3, 29 years old)

#### Preferences for antibiotic and antibiotic resistance information

Most participants expressed an interest in receiving more information about the indications for antibiotic use, the benefits, and short- and long-term effects of antibiotic use for themselves and their children. Some mothers particularly wanted to learn more about the causes of reproductive tract infections, for example leukorrhea, sexually transmitted infections and how to prevent and treat the infections.

*“I want to learn more about the causes of the reproductive tract infections such as leukorrea, sexually transmitted infections… and how to treat.”* (FGD#5 with mother, respondent#1, 26 years old)

The women also wanted to learn more on antibiotic use during pregnancy, delivery, and postpartum period.

*“I want to learn about the side-effects of antibiotics in general and during pregnancy in every period; for examples, delivery and after giving birth. I want to learn more on the definition of infectious diseases, which ones are infectious diseases and which ones are not*.” (FGD#3 with pregnant women, respondent#1, 39 years old)

Preferred sources of information were healthcare staff and village leaders. Other channels suggested by participants included television, radio, posters, websites and YouTube.

*“Source of information shall be provided by village speaker, Facebook, posters on roadside, hospitals, health centers, pharmacies, and village meetings.”* (FGD#4 with mother, respondent#7, 30 years old)

## Discussion

This qualitative study explored perceptions and reported practices of pregnant women and mothers with children under two regarding antibiotic use and resistance. All participants had some understanding of antibiotic use and resistance, but misconceptions were evident and contributed to inappropriate antibiotic use. These modifiable practices are likely to be due to relatively low levels of education amongst participants and lack of consistent messages within the different information sources women used to obtain information. Most participants felt antibiotics are an effective treatment but had concerns about short and long-term side-effects of antibiotic use during pregnancy and early infancy. While self-medication was reported, during pregnancy and when their children were under 2 years, women stated they relied more on allopathic doctors, regarding treatment, including antibiotics use. The desire to learn more and the intrinsic desire for their children to thrive are encouraging and can be leveraged to promote appropriate antibiotic use [13] .

As with previous studies [2, 13, 25, 27, 40], participants had a general understanding that antibiotic resistance was related to inappropriate or unnecessary antibiotic use, and that it makes the antibiotics less effective. However, there was a lack of clarity on what antibiotic resistance or unnecessary antibiotic use is. For instance, some participants thought it was the person who becomes resistant against antibiotics, and few could differentiate between bacteria and virus, or the importance of completing the prescribed dose. Reasons for early termination of antibiotic treatment may be due to concerns about side-effects or the cost, especially where people are not fully informed about correct antibiotic use of and risks associated with interrupting an antibiotic course [41–44] .

Pharmacies are often a first point of healthcare access in Lao PDR but are poorly regulated [23, 24, 32] and purchasing antibiotics from a pharmacy without prescriptions is common often due to cost convenience [41–44]. Most pregnant women in this study did not use antibiotics during their current pregnancy, and only took supplements (such as iron, folic acid and vitamin B1) provided during antenatal care visits. During pregnancy, women were more cautious about non-prescribed antibiotic use, preferring to seek advice from allopathic doctors. Concerns about non-prescribed antibiotic use during pregnancy related mainly to anxieties about negative effects on fetus, and the potential for congenital malformation. Other studies have identified that pregnant women are concerned about teratogenicity [45], especially where they have personal experience or know someone whose child was born with a disability due to real, or perceived, effects of antibiotic use during pregnancy [46].

In lower-middle income countries, younger children are often given medications by their parents without a doctor’s prescription [47]. The present study showed over half of the mothers incorrectly self-prescribed antibiotics to their children commonly to treat viral infections such as mild fever, cough, or sore throat, indicating a lack of understanding about antibiotic use. A previous study conducted in Lao PDR found that nearly half (46%) of the children under five years of age with diarrhea were given unprescribed antibiotics by their caregivers before presenting to the hospitals [48]. A study in China also reported that children under two years of age were given antibiotics for the treatment of fever (46%), dry cough (42%) and bronchitis (38%) [49].

The women in this study reported that during pregnancy and while seeking care for infants, they preferred to rely on the advice of allopathic doctors. As physicians and other healthcare workers play an important role in contributing to public knowledge of antibiotics, they are a powerful instrument for promoting appropriate antibiotic use and must prescribe appropriately and support patients to understand the importance of following the prescribed regimen. Health workers such as doctors and pharmacists can also educate people about the difference between bacterial and viral infections, and to better understand the concept of antibiotic resistance [12, 50, 51]. Pharmacists are also critical in supporting appropriate antibiotic use and providing correct guidance regarding their use. Self-medication with antibiotics is often associated with incorrect self-diagnosis, early termination of treatment and inappropriate choice of therapeutic class and dose. Comprehensive public information campaigns on more appropriate use of antibiotics targeting a variety of audiences such as the general public, healthcare workers and pharmacists can also contribute to reducing suboptimal antibiotic use. Academic institutions can educate future healthcare professionals on appropriate practice and effective patient consultation. They can also help developing tailored, educational interventions that could be used by healthcare workers during antenatal and postnatal care as well as through other mediums such as television and social media. Improved understanding of antibiotic use may promote restrictive attitudes toward antibiotics, contributing to reduced utilization or stronger adherence where antibiotics are justified, and thereby less antibiotic resistance.

The results of this study should be considered in the light of its limitations. As a qualitative study, caution should be taken in generalizing the findings beyond the sites where the data was collected. Nevertheless, the purpose of this exploratory study was not to make statistical inferences but to understand the cultural context of pregnant women and mothers’ perspectives of antibiotic use and resistance, providing a platform for further research. In addition, our study participants were mainly from Lao ethnic group and might not reflect the perspectives and experiences of women from different ethnic and cultural backgrounds within Lao PDR. Moreover, as the findings are based on the subjective experiences and self-reports of participants, the potential for recall and social desirability bias could not be discounted. Nevertheless, the study has provided useful information on perceptions and reported practices regarding antibiotic use and resistance among pregnant women and mothers of children under two. The findings gained from this study can be used to develop educational interventions (such as educational messages and campaigns) to promote appropriate antibiotic use.

## Conclusions

This study revealed that participants had misconceptions related to antibiotic use and resistance which might contribute to inappropriate antibiotic use. Participants, however, were concerned about antibiotic use during pregnancy and for their infants, and mostly sought advice from healthcare professionals in these contexts. Clear and consistent messages that can be disseminated using various channels, including trusted healthcare professionals, are likely to be the most effective. Messages can also potentially leverage the fundamental concern women have for their children. Awareness raising must also be complemented by efforts to address other determinants of inappropriate antibiotic use, including educating healthcare workers and pharmacists.

## Data Availability

The datasets generated and analysed during the current study are not publicly available due the nature of the qualitative dataset where it might be possible to identify the participants. However, data are available from the corresponding author on reasonable request.
